# Is Similia a Universal Law?

**Published:** 1892-10

**Authors:** J. R. Haynes

**Affiliations:** Indianapolis, Ind.


*Second Day—Morning Session.*

WEDNESDAY, June 22d, 10 A. M.

The following resolution was presented by Dr. W. P. Wesselhœft, and was adopted:

*Be it resolved*, That the sentiment and belief of this Association is that Dr. J. A. Biegler, as Chairman of the Board of Censors, has been untiring in his efforts for the best interests of the Association, and that the motion of Dr. George H. Clark, and seconded by many voices, was in no way intended to cast reflection upon him or upon his ability as the honored and respected Chairman of our Board of Censors, but solely to prevent a discussion of charges which were known in all their relations to every member of the Association, and upon which they had positive opinions, and which had found their way into the public press of the country.

The Auditing Committee reported that the accounts of the Secretary and Treasurer were correct and properly vouched for.

Report accepted and adopted.

---

### IS SIMILIA A UNIVERSAL LAW?

J. R. HAYNES, M. D., INDIANAPOLIS, IND.

There are between twelve thousand and fourteen thousand physicians in this country who claim to be homœopathists, and if a vote were to be taken upon the subject to-day as to whether it was a universal law and should be strictly adhered to in all medical cases, at least sixty per cent. would vote against it that there was no such a universal law, and that Homœopathy was only a system of medicine, and not the only true system of cure; that *Similia similibus curantur* should be written *Similia similibus curenter* (Hughes and numerous others), and that *Similia* would answer in some cases; but in others we claim the right to the armamentarium of all the schools of practice; and the physician who would not do so should not be allowed to practice, and be put down as a bigoted Hahnemanniac (the last pronounced with a slur). Now why is this the case? If you should speak to them about *The Organon*, many of them would not know what you meant; they would very likely ask is it some new medicine, or is it something good to eat, and who has gotten it up, or what is it made of, I have never heard of it before. Were you to tell them that it was Hahnemann's promulgation of the law of Homœopathy, and that no one could practice Homœopathy until he had made himself familiar with the law (the teachings of *The Organon*), and that it can be obtained nowhere else but from *The Organon*, they will laugh in your face and tell you that you are one of those new-fangled Hahnemanniacs, and that they have no use for it, that Hahnemann was an old fogy, that he may have done well enough in his day, but we are progressionists and have long since outgrown him, that we have learned a great many things that Hahnemann never knew or never thought of. No, I do not think that I want it.

Should this appear strange when *The Organon* has not been taught in any of the colleges, that it has been left out of their curriculum? Could anything else be expected when many of the professors of these misnamed colleges have never perused a page of *The Organon* in their lives? Then how could they teach it (the law) to their students; is there not something wrong here? Shall we allow it to continue?

In 1846, William Radde, of New York, published a small edition of *The Organon* which was translated by what we may call a syndicate to which C. Hering's name was attached as a sanction. This edition has long since been out of print. For the past thirty years any physician who may be so fortunate as to be the owner of a copy of it has, in most cases, kept it under lock and key, and refused to let any one take it from his office. I have known ten dollars offered for a copy and refused. Could it be expected that students attending the different colleges should supply themselves with such a rare work? And but few of them could afford it if it could be had at that price.

Is it strange, under these circumstances, that such a work is so little known? Have the I. H. A. members done their duty to them? Or should we be surprised at the number of homœopaths who are only such in name? Is there not a good excuse for them? Can we expect them to practice and believe a law that they have never heard, and which was to them a sealed book and which was entirely out of their reach?

In 1887 a new edition of *The Organon* was published by the publishing house of Bœricke, of Philadelphia, translated by C. Wesselhœft, which, as far as I can learn, has been a failure (*i. e.*, as to its sale, which has been very small), and for one I cannot say that I feel very sorry for its failure, for if it is a correct translation, then all of the other translations that I have seen are certainly incorrect.

Believing that the failure rests with the former, I do not feel like recommending that translation, and consequently there is no available edition of *The Organon* upon the market.

For the past year I have been trying to procure a sufficient number of subscribers to justify the publication of the translation made by Dr. Fincke of *The Organon*. I have sent out some four thousand circulars to the different homœopathic physicians, and so far I have about four hundred copies subscribed for. My reasons for so doing are that I believe Dr. Fincke's translation to be the one that is the nearest correct of any that have been made, and that I can recommend it to my friends, as well as to our young minor students who must in the order of nature come forward to fill our places in the profession and carry on the great work that was begun by Samuel Hahnemann. Shall it be done, or shall we let it prove a failure? Every one of you well know that *The Organon* is the life's blood of true Homœopathy, and without it Homœopathy must in the end prove a sad failure.

Hahnemann, in his *Organon*, says that there is a law of similia and that it is universal: *Organon*, §§ 26, 27, 28, 47, 48, 54, 70, 73, and numerous sections.

Shall we take Hahnemann's word for it, or shall we investigate the subject for ourselves? Shall we take his word for it, or shall we convert a belief into an actual knowledge by thoroughly investigating for ourselves? It has long passed a belief and become an actual knowledge. I have proved the efficacy of the law (*i. e.*, that there is such a law) hundreds of times, until it has long passed a belief and become an actual knowledge. I will venture the assertion that there is not one of the members of this I. H. A. who has not done the same and proved beyond a doubt that there is a law of similia and that it is universal.

You have administered the simillimum in acute cases and had your patients tell you in less than five minutes that they were better, and if the simillimum was not interfered with a rapid cure has followed.

Is not this enough to satisfy the most skeptical of the positive existence of the law and that it is immutable?

"Ye have Moses and the prophets, and if ye will not believe them, then ye would not though one rose from the dead."

### Discussion.

Dr. S. Long—Dr. Haynes is undoubtedly doing a good work in publishing Dr. Fincke's translation of *The Organon*, and I am glad that he has succeeded in getting a good many subscriptions at this meeting, most of the members taking from five to twenty copies. I think it is our duty to see him through.

Dr. Haynes—If every member of the profession were to obtain a copy of *The Organon* and use it as a Catholic does his prayer-book, reading it daily and studying it, he would make a far better doctor than he ever was before.

